# Editorial

**Published:** 1874-07

**Authors:** 


### Editorial.

Since our last issue, two events have occurred to disturb the calm monotony of the medical world. The Illinois State Medical Society has met, transacted, feasted, and adjourned. *Cui bono?* As a pleasant social gathering of representative men from different portions of this great State, assembled in compliance with the cordial invitation of their brethren of Chicago, to exchange friendly greetings, to renew the pleasant associations of past years, and to accept the hospitality of their municipal brethren, it was eminently a success. A more agreeable, genial, courteous and intelligent company of medical gentlemen has rarely ever graced with its presence a meeting of the State Medical Society. From a scientific standpoint this meeting cannot be regarded with so much satisfaction.

From a careful consideration of the meeting and its results, we are forced to the conclusion that the State Medical Society does not exercise the influence and authority in the profession which should be expected from its representative character and from the high order of intelligence of its members. One reason for this lack of influence is inherent in the organization of the Society, and is based upon what is popularly supposed to be an element of strength, but what has hitherto invariably been demonstrated to be an element of weakness in similar organizations; we mean its bulk and its consequent heterogeneity.

For the accomplishment of practical work for the attainment of scientific results, unity of purpose and spontaneity of effort are necessary, and these are scarcely attainable in so large a body of men, selected with reference rather to personal convenience than to professional fitness, and thrown together promiscuously.

The ratio of representation appears to be much too large, every local society having a minimum representation of one-fifth of its total membership, every college, two delegates, *i.e.*, about the same proportion, and every hospital, and every "*permanently organized medical institution of good standing*," *one*. If the basis of representation was designed to be numerical strength, the only objection to be made is its unnecessary magnitude, one-half or one-fourth of this number would be more than sufficient. If the basis, on the other hand, was designed to be professional efficiency and activity, then there appears to be an unjust discrimination against the schools. Moreover, in this same matter of representation, the largest hospital is placed upon the same footing as the smallest local dispensary with one physician and one patient, which may be "permanently organized and of good standing." Again, the mode of appointing standing committees is open to serious objection. Their organization, being intrusted to a nominating committee but imperfectly, if at all, acquainted with the members at large, with their qualifications or wishes, is necessarily effected in a disorderly and incongruous manner, so that the surgical expert is requested to report on obstetrics, and the student of physiology and psychological medicine upon diseases of children. The result of this confusion is, that neither the aspirant to reputation nor he having already attained thereto, will jeopardize his prospective or present fame by laboring in untried fields. Hence, reports, if made at all, are by no means up to the standard of excellence which they should, and would, under better management, attain.

Many years ago a member of the profession, noted as well for the sharpness of his satire as of his bistoury, remarked of a certain medical association, that it was simply a contrivance to enable Drs. —— and —— to hear themselves talk. This remark, justified, to a certain extent by the proceedings of the late meeting, has suggested the necessity for the application of a "gag law" to restrain the verbosity of certain individuals who have never yet learned to appreciate that while "speech is silvern, silence is golden."

It is to be hoped that the committee on publication will not deem it necessary to publish all that was said, for the enlightenment of our country brethren, but only that which had any meaning; the bulk of the "Transactions" will thus be greatly and advantageously reduced,—a "consummation devoutly to be wished."

What has been said concerning our State Medical Society will apply, *a fortiori*, to that more imposing (no sarcasm intended) body, the American Medical Association. Less talk, less personal advertising, less zeal for self, and more *esprit de corps*, and the advantages will soon be apparent in the increasing respect of the profession for that which should represent it as a body, and not simply its separate factions, schools, and societies.

In this Association, too, a reduction of the ratio of representation would apply with great advantage, until membership therein should come to be considered an honor, to be eagerly sought by eminent members of the profession, and not, as now, bestowed upon any willing to incur the expenditure of time and money necessarily incident to attendance on its meetings. H.

***Book-Keeping.***

A great favor would be conferred upon the Senior Editor of the JOURNAL, if the gentlemen who have done him the honor to borrow books from his library would take the opportunity of the mild summer weather to return them.

Particularly, just now, he would like to refer to vol. 28 of the *Am. Journal of the Med. Sciences* (1854); Beale's "How to Work with the Microscope;" "Hassall's Illustrations of Microscopic Anatomy," etc., and "Transactions of the Am. Med. Association," vol. viii.

Three hundred and odd volumes loaned (and lost) within the last three years, ought certainly to excuse him from further depredations. J. A. A.

***Declined.***

A letter concerning a "pretended *analasys*" "of the Iodo-Bromide of Calcium Comp." is respectfully declined—inasmuch as it is unintelligible. If the Comp. referred to be as intricate as the orthography and syntax of the letter, the "*analasys*" must be "a fraud," and the Comp. itself, like one of Lord Dundreary's conundrums, "one of those things that no fellow can find out, you know."

Concerning this, or any other similar compound, we have only to say, that whatever may be their merits or demerits, whether they possess all the value claimed for them by their proprietors, or are, on the other hand, utterly valueless, no apology can be made for physicians who use them. The prescription of proprietary medicines by physicians involves not only a breach of professional confidence toward the patient, who has a legal and moral right to expect from his physician the use of his own best skill and judgment, both in the diagnosis of his malady and in the application of remedies for its relief; but it involves also a total abandonment of the whole field of therapeutics to the manufacturing druggist, who is, in all probability, totally ignorant of its first principles. We make no complaint against the proprietors or venders of these nostrums, of which the manufacture and sale, like all other commercial enterprises, will be governed by the law of supply and demand. We do not propose to go outside of our own profession to find the sources of abuses within it. The evil lies within our own precincts. Were there no demand, there would be no supply. Manufacturers would never waste a dollar in the preparation of nostrums which physicians would not use.

The times have changed, and for the worse. Formerly, the popular mind was the object of the delusive arts of quackery, and the ignorant masses were beguiled with the watchword, "every man his own doctor." But the popular mind is fickle, and success based upon any of its phases, expensive in its attainment and transitory in its duration; hence, this unstable basis of commercial enterprise was abandoned for a higher or more secure, if more restricted foundation, the demoralization of the medical profession—for such will surely be the result of the abandonment of the field of therapeutics to the nostrum manufacturers and peddlers.

H.

***The County Hospital.***

The County Commissioners have at last selected and purchased a site for the proposed County Hospital. In one respect, at least, they have displayed wisdom and good judgment, *i. e.*, in securing ample room for the necessary buildings and grounds. We say buildings, as, after having demonstrated hitherto their appreciation of the necessities of such an institution, it is hardly supposable that they could relapse so far into the abyss of ignorant and old-fashioned prejudice, as to ignore the teachings of modern sanitary science, and erect a cumbrous pile of brick and stone towering to the skies, to become in a few years what no General Hospital should ever be, a pest house.

Mistakes enough have been made in the construction of every building heretofore erected for hospital purposes in this city—

mistakes, whose consequences cannot be averted by the most scrupulous care, the most rigid sanitary system. Let the commissioners, like wise men, profit by the experience of others, not, like fools, buy their own experience at a heavy cost of money and human life.

The opportunity is now offered to the commissioners to immortalize themselves, to confer a great blessing on their fellow citizens and on humanity at large, by erecting a hospital fully up, in all respects, to the requirements of modern sanitary science; that our city, which has led the van in the march of improvement in so many directions, may still keep her accustomed place at the head in this direction also.

Mediæval castles, piled story upon story skyward, with loophole windows and flying buttresses to exclude both light and air, with winding stairs and vaulted corridors, turrets and pinnacles, are not needed for the safe custody and care of the sick—but long, broad, lofty, well lighted and ventilated wards or pavilions, with light and air above, beneath, and on all sides, raised above the earth sufficiently to secure dryness and avoid dampness, and yet not so high as to require many steps for ingress and egress, built with sufficient solidity to secure necessary strength, and yet so cheaply as to justify the destruction of any one pavilion so soon as the increase in the mortality within its walls shall indicate that its atmosphere has become vitiated.

Nor should æsthetics be altogether ignored. The construction of a central building for the administrative department of the institution, will admit of any degree of beauty of design, of solidity of structure, and of elaborateness of ornamentation. As this should be a permanent building, in this, and in the ornamentation of the grounds, the architect and landscape gardener should have full scope for the exercise of their genius and taste. But the pavilions for the sick should be constructed with an eye single to the attainment of the most perfect sanitary conditions, in the light of the most advanced sanitary science.

In considering plans for the new County Hospital, let our commissioners bear in mind one radical idea which should underlie any plan for such an institution, the idea of a wheel, with the permanent administration building represented by the hub, the pavilions for the sick by the spokes, the enclosure by the rim, and ornamental grounds by the spaces between the spokes. This or some modification of this idea has been demonstrated by modern experience to be that most advantageously applicable to plans for hospitals—whether the best sanitary results or the most economical administration be the governing principle.

A prominent member of the surgical staff of one of our largest hospitals has recently stated publicly, that the mortality amongst surgical cases in the local hospitals was appreciably larger than under similar conditions in private practice. Let us hope that the new County Hospital, constructed upon correct sanitary principles, will turn the scale of comparative mortality in the other direction. H.

***The American Journal of Obstetrics and Diseases of Women and Children.***

We publish in another column,* by request, the valedictory of Dr. B. F. Dawson on his withdrawal from the editorial chair of this Journal, in which a few of the perplexities and difficulties in the life of a medical journal are detailed. Dr. Dawson has signalized his retirement by an act as graceful as it is liberal, and most worthy of imitation, *i. e.*, the establishment of an annual prize of one hundred and fifty dollars, gold, for the best essay on the Congenital Deformities and Diseases depending on Maladies of the Uterus or Membranes.

If Dr. Dawson's successor is actuated by the same spirit which has been manifested by the founder of the work, the *American Journal of Obstetrics* will always be assured of the same measure of success which has marked the past. " May it live long and prosper." H.

***Fiat Justitia, ruat Cœlum.***

We publish also, by request, a circular from the Secretary of the Clinton Co. (Iowa) Medical Society, announcing the expulsion of two of its members for violation of its by-laws. Without presuming to express any opinion as to the justice of the act, we must take the liberty to say, that it is positively refreshing to find a medical society having the moral courage to expel obnoxious individuals from its midst. On general principles we say, well done!

* Crowded out.

***Sanitary.***

We take pleasure in calling attention to the favorable sanitary condition of the city, as compared with corresponding periods in former years, demonstrated by the tabulated reports of the Board of Health, endorsed officially by the Sanitary Superintendent, Ben. C. Miller. The possibility of preserving the health of the city, by diligent and judicious enforcement of sanitary laws, having been demonstrated, there will be no excuse for a relapse into the former slough of ignorance, dirt, and disease.

